# Plants from Brazilian Cerrado with Potent Tyrosinase Inhibitory Activity

**DOI:** 10.1371/journal.pone.0048589

**Published:** 2012-11-16

**Authors:** Paula Monteiro Souza, Silvia Taveira Elias, Luiz Alberto Simeoni, José Elias de Paula, Sueli Maria Gomes, Eliete Neves Silva Guerra, Yris Maria Fonseca, Elton Clementino Silva, Dâmaris Silveira, Pérola Oliveira Magalhães

**Affiliations:** 1 Department of Pharmaceutical Sciences, School of Health Sciences, Campus Darcy Ribeiro, University of Brasília, Brasília, Brazil; 2 Department of Odontology, School of Health Sciences, Campus Darcy Ribeiro, University of Brasília, Brasília, Brazil; 3 Department of Botany, Institute of Biological Science, Campus Darcy Ribeiro, University of Brasília, Brasília, Brazil; Gentofte University Hospital, Denmark

## Abstract

The increased amount of melanin leads to skin disorders such as age spots, freckles, melasma and malignant melanoma. Tyrosinase is known to be the key enzyme in melanin production. Plants and their extracts are inexpensive and rich resources of active compounds that can be utilized to inhibit tyrosinase as well as can be used for the treatment of dermatological disorders associated with melanin hyperpigmentation. Using *in vitro* tyrosinase inhibitory activity assay, extracts from 13 plant species from Brazilian Cerrado were evaluated. The results showed that *Pouteria torta* and *Eugenia dysenterica* extracts presented potent *in vitro* tyrosinase inhibition compared to positive control kojic acid. Ethanol extract of *Eugenia dysenterica* leaves showed significant (*p*<0.05) tyrosinase inhibitory activity exhibiting the IC_50_ value of 11.88 µg/mL, compared to kojic acid (IC_50_ value of 13.14 µg/mL). *Pouteria torta* aqueous extract leaves also showed significant inhibitory activity with IC_50_ value of 30.01 µg/mL. These results indicate that *Pouteria torta* and *Eugenia dysenterica* extracts and their isolated constituents are promising agents for skin-whitening or antimelanogenesis formulations.

## Introduction

Tyrosinase (polyphenoloxidase, PPO, E.C. 1.14.18.1) is a copper containing enzyme that catalyzes two distinct reactions, involving molecular oxygen with various phenolic substrates: the o-hydroxylation of monophenols to o-diphenols (monooxygenase or cresolase activity) and the subsequent oxidation of o-diphenols to o-quinones (diphenolase or catecholase activity) [1], [2]. In melanin biosynthesis, tyrosinase converts L-tyrosine, monophenol, firstly to L-DOPA (o-diphenol) and this to o-dopaquinone, which is spontaneously cyclated in form of leukodopachrome and quickly converted into dopachrome, which polymerizes and form melanin [1], [3], [4].

Melanin is one of the most widely distributed pigments and is found in bacteria, fungi, plants and animals. The color of mammalian skin and hair is determined by a number of factors, the most important of which is the degree and distribution of melanin pigmentation [5]. Melanin plays a crucial role in the absorption of free radicals and protects skin from various types of ionizing radiations, including UV [6]. However, the increased amount of melanin results in pigmentary skin disorders and occurs as a result of both genetic and enviromental factors [7], [8].

Various dermatological disorders, such as melasma, age spots and sites of actinic damage, arise from the accumulation of an excessive level of epidermal pigmentation [5]. Tyrosinase inhibitors therefore can be clinically useful for the treatment of some dermatologic disorders associated with melanin hyperpigmentation and find applications in cosmetic products for whitening and depigmentation after sunburn [9], [10]. Despite the extensive researches on lightening agents and hyperpigmentation, the existing agents present limitations in term of high toxicity, low stability, poor skin-penetration, and insufficient activity [11]. Several compounds, such as the well-known tyrosinase inhibitors, hydroquinone, kojic acid, arbutin and corticosteroids, can cause adverse reactions, such as dermatitis and skin irritation, melanocyte destruction, post-inflammatory pigmentation, ochronosis, cytotoxicity and skin cancer [12]. Therefore, many tyrosinase inhibitors that suppress melanogenesis have been actively studied with the aim of developing preparations for the treatment of hyperpigmentation [5], [6], [13].

Cerrado is the second biggest Brazilian biome and supports a wide variety of species. Plants from cerrado are known as source of compounds of high biotechnological interest, with applications on medical and food industries [14] . Additionally, several plants of families found in this biome, e.g. Apocynaceae, Sapotaceae, Fabaceae, among others, have interesting biological activities, such as antimicrobial, anti-inflammatory, and antifungical [15]–[17]. Due to the rich plant diversity existing in cerrado, it is very encouraging to explore the potential of cerrado plants for dermatologic purposes; nonetheless, this biome has been poorly studied to evaluate the efficacy and therapeutic effects of crude extracts or isolated compounds [18].

The overall goal of this study is to screen the selected cerrado plants for new tyrosinase inhibitors using in vitro assays, which may offer a new effective and safe therapeutic approach in the management of dermatologic disorders associated with melanin hyperpigmentation.

## Materials and Methods

### Chemicals and Reagents

The following chemicals were obtained from Sigma-Aldrich: Tyrosinase from mushroom (lyophilized powder, ≥1000 unit/mg solid), L-tyrosine (≥98%) and kojic acid.

### Plant Material

The plant material was collected from the cerrado biome in Brasília and surroundings. Botanical identification was performed by Professors José Elias de Paula and Sueli Maria Gomes. The voucher specimens were deposited at Herbarium of the University of Brasilia (UB) and Herbarium of the University of Campinas (UEC) (Table 1). All necessary permits were obtained for the described field studies.

**Table 1:** Crude extracts tested against tyrosinase.

Plant species	Part of plant tested (solvent)	Voucher number
**Apocynaceae**		
*Allamanda blanchetti* A.DC.	1L(e,h), 2S(e), 3F(e,h)	(UEC) 142021
*Hancornia speciosa* Gomes	L(e,h)	(UEC) 142204
*Tabernaemontana solanifolia* A.DC.	L(e,h)	(UB) 487
**Myrtaceae**		
*Eugenia dysenterica* DC.	L(a,e,h)	(UB) 914
**Fabaceae**		
*Stryphnodendron adstringens* (Mart.) Coville	4SB(e)	(UB) 911
**Rubiaceae**		
*Genipa americana* L. Var. caruto (H.B.K) K. Shum.	L(e,h), F(e,h), 5P(e)	(UB) 915
**Sapotaceae**		
*Pouteria gardneri* (Mart. & Miq.) Baehni	L(e,h)	(UB) 3672
*Pouteria ramiflora* Radlk.	L(a,e,h)	(UB) 3671
*Pouteria caimito* Radlk.	L(a,e,h)	(UB) 27284
*Pouteria torta* Radlk.	L(a,e,h), F(e), P(e)	(UB) 3674
**Caryocaraceae**		
**Caryocar** cf. **villosum** (Aubl.) Pers.	F(e)	(UB) 907
**Sapindaceae**		
*Sapindus saponaria* L. variedade *inaequalis* (DC.) Radlk.	F(e)	(UB) 916

1 L: leaf;

2 S: stem;

3 F: fruit;

4 SB: stem bark;

5 P; peel. Crude extract: (e) ethanol; (h) hexane; (a) aqueous.

### Extraction

The plant material was dried at room temperature and powdered in a knife mill. The hexane and ethanol crude extracts were obtained in the following way: plant material (40 g) was macerated at room temperature for seven days (repeated for three times), first with hexane (2 L), followed by ethanol (2 L). After filtration, the solvents were removed under reduced pressure at temperatures below 40°C. The aqueous extract from 400 g of plant material was obtained by infusion, using distillated water (3 L). After filtration, water was removed by lyophilization. To process the *in vitro* assays, no previous treatment was used over crude extracts.

### HPLC Analysis

*Pouteria torta* and *Eugenia dysenterica* aqueous extracts were analyzed using LaChrom Elite HPLC system (Hitachi, Tokyo, Japan) liquid chromatograph equipped with L2130 pump, L2200 auto-sampler; L2300 column oven was set at 25°C and a L2455 DAD detector (Hitachi, Tokyo, Japan). The detector was set at 280 nm. Separation was performed by Purospher Star reverse phase C18e column (5 µm, 150 mm×4.6 mm i.d.) in combination with an appropriate guard column (4×4; 5 µm particle size) (Merck, Germany). The mobile phase was a linear solvent gradient system consisting of phosphoric acid (1%) (A) and CH_3_CN (B), at a flow rate of 0.6 mL/min. Data acquisition was performed using EZChrom Elite software (version 3.3.2 SP1 (Scientific Software. Inc.). The compounds present in the extract were characterized according to their UV–Vis spectra and identified by their retention times in comparison with those of commercial standards.

### Inhibition of Tyrosinase Activity

Tyrosinase inhibition assay was performed using Khatib et al. (2005) method with modifications [19]. Sodium phosphate buffer (60 µL, 50 mM) at pH 6.5, 30 µL tyrosinase (25 U/mL) and 10 µL of the plant extract (1 mg/mL) were inserted into 96-well plates. The hexane and ethanol extracts were dissolved in DMSO (Dimethyl Sulfoxide) and the aqueous extracts were dissolved in distilled water. After 5 min of incubation at room temperature, 100 µL L-tyrosine (2 mM) were added and incubated for additional 20 min. The optical density (OD) of the samples at 475 nm (BioTek Synergy HT Multi-Mode Microplate Reader) were measured compared to control without inhibitor, demonstrating a linear color change with time during the 20 min of the experiment. Control incubations represent 100% enzyme activity and were conducted in a similar way by replacing extracts by buffer. For blank incubation, to allow for absorbance (A) produced by the extract, the enzyme solution was replaced by buffer. The inhibitory activity was determined by comparing the enzyme activity in the absence and presence of the evaluated inhibitor. Kojic acid was used as positive control.

### Cytotoxicity Assay

The cell lines, one human keratinocyte cell line (HaCat), and one fibroblast cell line (L-929), were grown as monolayers in a mixture of Dulbecco’s modified Eagle medium and supplemented with 10% fetal bovine serum and 1% antibiotics (penicillin-streptomycin). Cells were maintained at 37°C and 5% of CO_2_. For all experiments, cells were detached with trypsin (0.25%)/EDTA (1 mM) solution. All cell culture reagents were purchased from Sigma-Aldrich (ET. Louis, MO). The cells line used are described in the ATCC (American Type Culture Collection).

The cells were seeded at the density of 5×10^3^ cells/well in a 96-well plate and then treated with extracts at 500 µg/mL and with extracts at IC_50_ values. For negative control cells were treated only with the correspondent solvent used to dilution off extracts. Following 24 after extracts’ treatment, cell death was assessed by MTT assay and the absorbance was measured at 570 nm in a Beckman Counter reader. This test assesses the ability of mitochondrial enzymes off cells treated to convert tetrazolium salts (MTT) in formazan, so only viable cells have the ability to do this reduction, or cells which have not undergone sufficient to reduce its toxicity mitochondrial activity, and then the absorbance measured correspond to viable cells. For cytotoxicity assay, we have used the extracts that presented best results at tyrosinase inhibition assay. All experiments were carried out at least three independent times and were performed in triplicates.

### Statistical Analysis

All experiments were carried out in triplicate and data are expressed as mean ± SD (Standard Deviation). The enzyme inhibitory activity was calculated using the following formula: % Inhibition = [(C−A)/C]×100, where C represents the absorbance of the enzyme activity and contains enzyme and substrate; and A represents the absorbance of the test and contains enzyme, plant extract, and substrate. Any increase in absorbance due to the spontaneous hydrolysis of substrate or to rule out unspecific enzymatic inhibition was corrected by subtracting the rate between the samples and the blank incubation.

The half maximal inhibitory concentration (IC_50_) is a measure of the effectiveness of a compound in inhibiting biological or biochemical function values. In this study, the IC_50_ were estimated by nonlinear regression analysis. The dose-response curve was obtained by plotting the percentage inhibition versus logarithm of the extract concentration. For IC_50_ determination of plant extracts against tyrosinase, a dose-response curve with 12 values of concentration (1000 - 0.48 µg/mL) was employed. The Student’s t-test was applied to assess the presence of significant differences (*p*<0.05) between the extract and the positive control. All the statistical analyses were accomplished using the computer software GraphPad Prism Version 5.0.

To cytotoxicity assay, statistical analysis was performed on the means of the triplicates at least three independent for all experiments using GraphPad Prism Version 5.01, applying ANOVA One-way and Tukey’s multiple comparison.

## Results and Discussion

Melanogenesis is a physiological process resulting in the synthesis of melanin pigments, which play a crucial protective role against skin photocarcinogenesis. Alterations in melanogenesis may be responsible for some clinical and histopathological features of dermatologic disorders associated with melanin hyperpigmentation. Tyrosinase inhibitors may be clinically used for the treatment of some skin disorders associated with melanin hyperpigmentation and are also important in cosmetics for skin whitening effects. Therefore, several chemicals from plant origin have been tested as cosmetics and pharmaceuticals to prevent overproduction of melanin in epidermal layers or as whitening agents [4], [6], [20].

**Figure 1:**
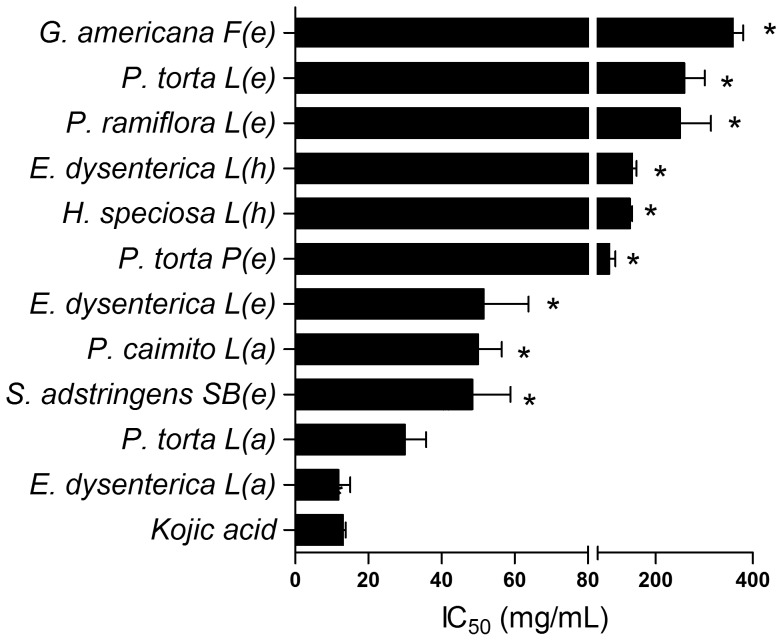
IC_50_ values of tyrosinase inhibition assay. Kojic acid as positive control. *p<0.05 *vs* Positive control. L: leaf; F: fruit; SB: stem bark; P: peel. Crude extract: (e) ethanol; (h) hexane; (a) aqueous.

Thirteen cerrado plant species were selected for investigation, and 33 extracts were tested for tyrosinase inhibitory activity. The results are shown in Table 2. Some plant extracts which demonstrated a significant capability to inhibit tyrosinase are described for the first time for this biological property. This study revealed that 22 from 33 extracts present a poor tyrosinase inhibitory activity (less than 65%) compared to the positive control kojic acid (81.31%). Among the screened plants, the most active extracts tyrosinase belonged to *Pouteria* species as well as *Eugenia dysenterica* and *Stryphnodendron adstringens*. Some species, such as *Hancornia speciosa* and *Genipa americana*, presented weak activity on tyrosinase. Kojic acid was used as a standard tyrosinase inhibitor and showed the IC_50_ value of 13.14 µg/mL. Figure 1 shows the IC_50_ values of the extracts that present high inhibition on tyrosinase activity.

**Table 2:** Evaluation of the potential activity of 33 crude extracts on tyrosinase.

Species	Part of plant (solvent)	Inhibition (%)
*Allamanda blanchetti*	1L(e)	19.48±0.03
	L(h)	24.1±0.02
	2S(e)	NI
	3F(e)	9.3±0.08
	F(h)	10.24±0.04
*Hancornia speciosa*	L(e)	36.91±0.05
	L(h)	79.92±0.01
*Tabernaemontana solanifolia*	L(e)	22.5±0.00
	L(h)	18.53±0.05
*Caryocar villosum*	F(e)	32.32±0.08
*Stryphnodendron adstringens*	4SB(e)	95±0.03
*Eugenia dysenterica*	L(a)	90.47±0.09
	L(e)	100±0.08
	L(h)	100±0.08
*Genipa americana*	5P(e)	30.44±0.14
	L(e)	30.44±0.10
	L(h)	12.44±0.02
	F(e)	73.16±0.03
	F(h)	29.88±0.02
*Pouteria caimito*	L(a)	87.6±0.05
	L(e)	NI
	L(h)	27.95±0.01
*Pouteria gardneri*	L(e)	26.67±0.08
	L(h)	15.58±0.02
*Pouteria ramiflora*	L(a)	28.37±0.32
	L(e)	79.77±0.05
	L(h)	19.8±0.02
*Pouteria torta*	L(a)	100±0.07
	L(e)	95.39±0.05
	L(h)	14.65±0.02
	F(e)	29.32±0.07
	P(e)	63.46±0.11
*Sapindus saponaria*	F(e)	25±0.03
Kojic acid		81.31±0.01

1 L: leaf;

2 S: stem;

3 F: fruit;

4 SB: stem bark;

5 P; peel. Crude extracts: (e) ethanol; (h) hexane; (a) aqueous. NI: no inhibition. Results are represented by mean of inhibition at concentration 1000 µg/mL.

* Positive control for tyrosinase tests.

The aqueous, ethanol and hexane extracts from leaves of *E. dysenterica* showed high inhibitory activity on tyrosinase (inhibition of 90.5%, 100% and 100%, respectively). Among these extracts, the aqueous extract was the most potent inhibitor with with IC_50_ values of 11.88 µg/mL, when compared to kojic acid. The ethanol and hexane extracts exhibited a moderate activity against tyrosinase, with IC_50_ values of 51.54 and 151.37 µg/mL, respectively, differing from *Myrcia sphaerocarpa*, belonging to the same family of *E. dysenterica* (Myrtaceae), previously reported presenting very low inhibition (2%) [20]. In another research, flowers of *Eugenia caryophyllata* also showed a weak inhibitory activity (12%) [21]. However, essential oil from *E. dysenterica* leaves presents linalool [22], that showed inhibitory activity of 50.5% on tyrosinase [23]. Thus, for *E. dysenterica* this is the first report about such activity.

The genus *Pouteria* extracts showed more potent inhibitory activity compared to kojic acid. The extracts from *P. torta*, *P. ramiflora* and *P. caimito* leaves showed 79–100% inhibitory activity on tyrosinase. Aqueous extract from leaves of *P. torta* revealed inhibitory properties on enzyme with IC_50_ values of 30.01 µg/mL. A weak activity was found for the ethanol extracts from fruit peel and leaves of *Pouteria torta* with IC_50_ value of 104.34 and 258.53 µg/mL, respectively, against tyrosinase. The ethanol extract from leaves of *P. ramiflora* had little effect on the tyrosinase activity (IC_50_ value of 249.83 µg/mL) and a good inhibition was found for leaves of *Pouteria caimito* aqueous extract with IC_50_ value of 50.01 µg/mL against enzyme. No significant inhibitory activity was shown by the others extracts of genus *Pouteria*. Previous studies reported the ability of some Sapotaceae species to inhibit tyrosinase. Momtaz et al. (2008) reported that methanol and acetone extracts of the stem bark from *Sideroxylon inerme* showed significant inhibition of monophenolase activity (IC_50_ values of 63 µg/mL and 82 µg/mL, respectively) [24]. Isolated compounds from other species of Sapotaceae family, *Synsepalum dulcificum*, showed inhibitory activity against tyrosinase [25]. As far as we know, no tyrosinase inhibitory activity had been reported for *Pouteria* species, which may be a new source of inhibitors for the treatment of hyperpigmentation.

The ethanol extract of *Stryphodendron adstringens* (Fabaceae) bark stem also presented inhibitory activity on tyrosinase (inhibition of 95%) with IC_50_ value of 48.45 µg/mL. The aqueous extract from bark stem and seeds of *S. adstringens* previously showed strong activity on tyrosinase with 52% and 90% inhibition, respectively [20].

In the experimental conditions, the evaluated Apocynaceae species did not exhibit significant inhibition activity on tyrosinase. The hexane extracts from *Hancornia speciosa* leaves inhibited tyrosinase (79.92%) with weak effect (IC_50_ of 146.60 µg/mL). Also a low inhibition against tyrosinase was found for the hexane extract of *Genipa americana* (Rubiaceae) fruit with IC_50_ value of 361.23 µg/mL. One research reported that the extract of *G. americana* bark exhibited weak activity against tyrosinase with 23% inhibition [20].

**Figure 2:**
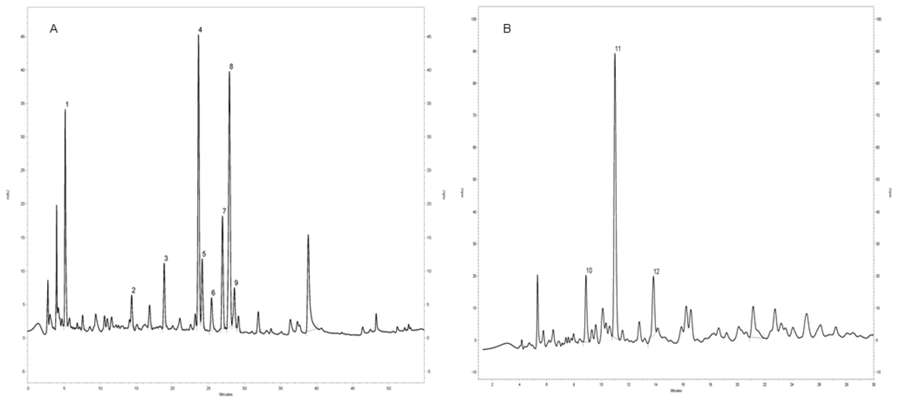
HPLC/DAD chromatogram of aqueous leaf extract of *P. torta* (A) and *E. dysenterica* (B).

**Figure 3:**
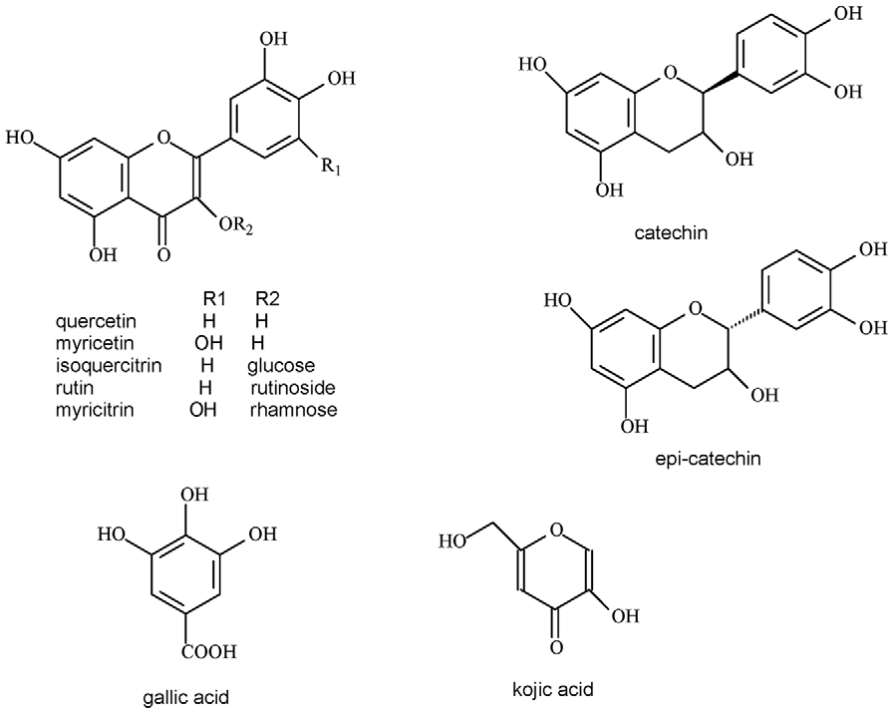
Identified compounds.

The chromatography profile showed that aqueous leaf extract of *Pouteria torta* presents a large number of compounds (Figure 2A). It was observed nine main peaks. The peak 1 has characteristic UV/Vis spectra of gallic acid derivatives. In addition, the peaks of 2 and 3 have characteristic UV/Vis spectra of catechin derivatives, with maximum absorbance in close to 280 nm, and no absorption at 320 or 350 nm. Moreover, the peaks 4–9 have characteristic UV/Vis spectra of flavonols, with λ_max_ between 340 and 370 nm. It was possible to identify catechin (peak 2), epicatechin (peak 3), myricitrin (peak 4), rutin (peak 7) and isoquercitrin (peak 8) by comparison of commercial standards (Figure 3).

Aqueous leaf extract of *Eugenia dysenterica* showed three main peaks in HPLC/DAD chromatogram (Figure 2B). The peaks 10–12 have characteristic UV/Vis of catechin derivatives, with maximum absorbance in close to 280 nm, and no absorption at 320 or 350 nm. These results corroborate with described by Arapitsas (2008) that showed the characteristic UV/Vis spectra of catechin and flavonoids.

From *P. torta* leaves aqueous extract was isolated myricitrin (myricetin-3-O-rhamnoside) as majority compound (forthcoming paper). Myricetin, as well myricitrin in minor degree, showed inhibitory activity over tyrosinase [26], [27]. In the same way, gallic acid and catechins derivatives also are described as tyrosinase inhibitors [28], [29]. The presence of these compounds can explain, at least in part the activity of aqueous extract of *P. torta*, as well as *E. dysenterica*.

The mechanism of mushroom tyrosinase inhibition by flavonols and catechins is well described on literature [30], [31] and in some cases, are very similar to kojic acid inhibition mechanism. Some phenolic compounds, as flavonols and catechins, can present a good affinity for the enzyme. These compounds, containing 3-hydroxy-4-keto, 5-hydroxy-4-keto and/or di-hydroxyl moiety can chelate cooper at the active site of the enzyme, thus preventing dopachrome formation in a competitive way [31], [32].

Kubo and cols (2007), in an elegant experiment, proposed a mechanism to explain the oxidative degradation of quercetin catalyzed by mushroom tyrosinase [33]. According this mechanism, quercetin is oxidized to corresponding *o*-quinone and subsequent isomerized to *p*-quinone methide form. Addition of water led to formation of protocatechuate intermediary, which is oxidized to a corresponding *o*-quinone [33].

**Figure 4:**
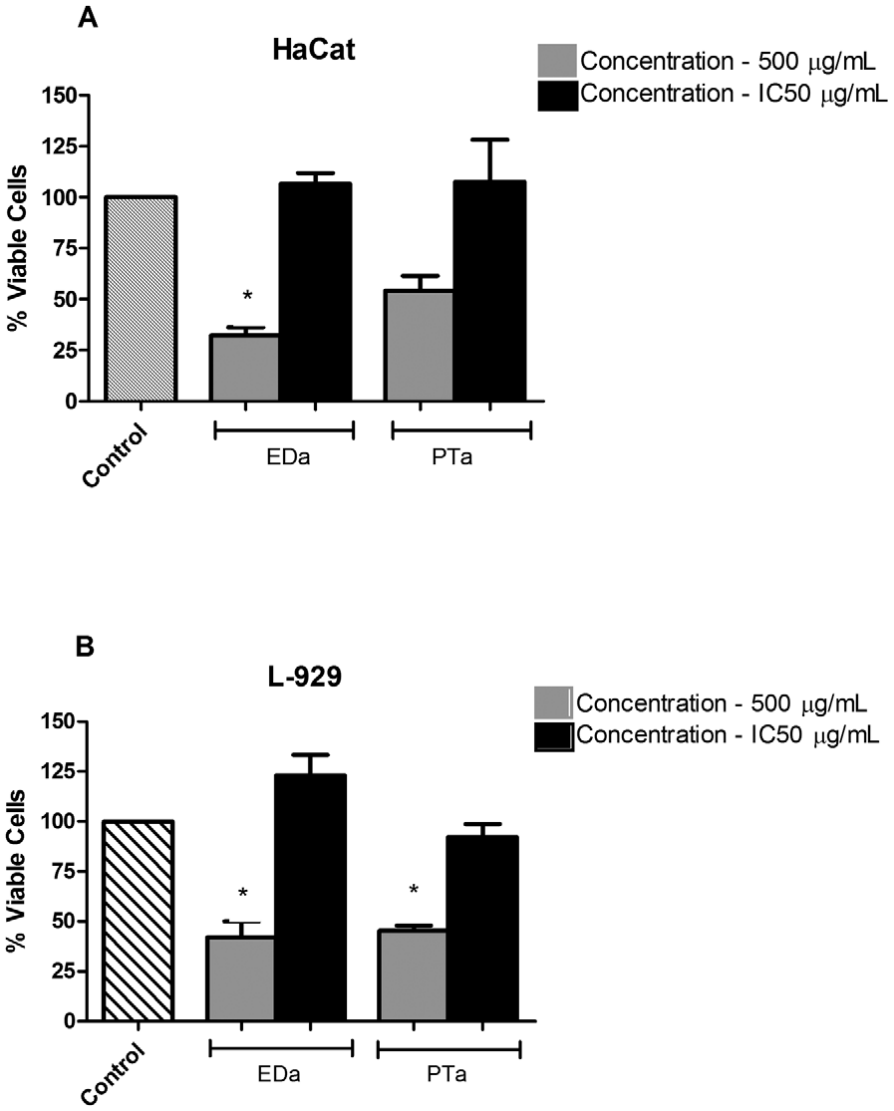
Cytotoxicity assay of keratinocyte (HaCat) and fibroblast (L-929) cell lines. Cells were treated with extracts at the concentration of 500 µg/mL and IC50 value. Results depict average of three independent experiments, each performed in triplicate. Treatment was conducted for 24 h. Control was normalized to 100%. *p<0.05 *vs* control. Crude extracts: EDa- *Eugenia dysenterica (*aqueous); PTa - *Pouteria torta (*aqueous).

The cytotoxic effect of aqueous extract from leaves of *Eugenia dysenterica* and *Pouteria torta* were evaluated in two cell lines, keratinocyte and fibroblast, which cells take part of skin components. Treatment with *Eugenia dysenterica* and *Pouteria torta* at IC_50_ value concentration (11.88 µg/mL and 30.01 µg/mL, respectively) did not result in cell death in both cell lines, in comparison to control, after 24 hours of treatment. Furthermore, these extracts induced mild proliferation in both cell lines. In the HaCat cell line, *Eugenia dysenterica*, at the concentration of 500 µg/mL, inducing cell toxicity, resulting in 32.1% of live cells after 24 hours of treatment (p<0.05), and *Pouteria torta* caused cell death, but the cytotoxic effect did not present significant differences compared to control, showing that the extracts did not present a severe cytotoxic at high level of extract’s concentration (Figure 4A). In the L-929, both extracts, at the concentration of 500 µg/mL, inducing cell toxicity, resulting in 41.9.1% and 45.3% of live cells after 24 hours of treatment (p<0.05) (Figure 4B).

### Conclusion

The extracts of plants from Brazilian Cerrado were screened for potential inhibitory activity on tyrosinase. The results indicated that *Pouteria torta* and *Eugenia dysenterica* extracts present highest activity on tyrosinase than kojic acid. Cytotoxicity of *E. dysenterica and P. torta* extracts also were investigated and our data showed that all evaluated extracts were not cytotoxic to both HaCat and L-929 cells at IC_50_ value concentration. Additionally, taken together our results suggest that *Eugenia dysenterica and Pouteria torta* have potential to be used as topic formulation in skin without causing any cytotoxic effect. These extracts are only cytotoxic at higher concentrations that are not common using in formulations. Furthermore, we can prove that these extracts are not cytotoxic to keratinocyte and fibroblast at IC_50_ value concentration.

In conclusion, based on the obtained results from tyrosinase inhibitory assays, we report here for the first time that extracts of *Pouteria torta* and *Eugenia dysenterica* present potential to be used in skin-whitening or antimelanogenesis preparation for cosmetics or therapeutic purposes. The identification of active constituents responsible for the anti-melanogenesis effect and the phytochemical profiling are currently underway.
